# Risk Factors for and Impact of Pre‐Engraftment Syndrome on Outcomes Following Single‐Unit Cord Blood Transplantation in Adults

**DOI:** 10.1002/ajh.70094

**Published:** 2025-09-26

**Authors:** Masatoshi Sakurai, Keisuke Kataoka, Kota Mizuno, Takuto Mori, Shuichi Shirane, Hirotoshi Sakaguchi, Takehiko Mori, Masatsugu Tanaka, Masahito Tokunaga, Makoto Onizuka, Mamiko Sakata‐Yanagimoto, Jun Ishikawa, Yuta Katayama, Shuichi Ota, Masashi Sawa, Jun Kato, Yuta Hasegawa, Koichi Onodera, Norimichi Hattori, Shigesaburo Miyakoshi, Nobuyuki Takayama, Tetsuya Nishida, Koji Kato, Fumihiko Ishimaru, Yoshiko Atsuta, Junya Kanda, Hideki Nakasone, Seitaro Terakura

**Affiliations:** ^1^ Division of Hematology, Department of Medicine Keio University School of Medicine Tokyo Japan; ^2^ Division of Molecular Oncology National Cancer Center Research Institute Tokyo Japan; ^3^ Department of Hematology, Graduate School of Medicine Kyoto University Kyoto Japan; ^4^ Division of Cancer Evolution National Cancer Center Research Institute Tokyo Japan; ^5^ Department of Hematology Juntendo University School of Medicine Tokyo Japan; ^6^ Department of Advanced Hematology Juntendo University School of Medicine Tokyo Japan; ^7^ Children's Cancer Center National Center for Child Health and Development Tokyo Japan; ^8^ Department of Hematology Institute of Science Tokyo Tokyo Japan; ^9^ Department of Hematology Kanagawa Cancer Center Yokohama Japan; ^10^ Department of Hematology Imamura General Hospital Kagoshima Japan; ^11^ Department of Hematology/Oncology Tokai University School of Medicine Isehara Japan; ^12^ Department of Hematology University of Tsukuba Hospital Tsukuba Japan; ^13^ Department of Hematology Osaka International Cancer Institute Osaka Japan; ^14^ Department of Hematology Hiroshima Red Cross Hospital & Atomic‐Bomb Survivors Hospital Hiroshima Japan; ^15^ Department of Hematology Sapporo Hokuyu Hospital Sapporo Japan; ^16^ Department of Hematology and Oncology Anjo Kosei Hospital Anjo Japan; ^17^ Department of Hematology Hokkaido University Hospital Sapporo Japan; ^18^ Department of Hematology Tohoku University Hospital Sendai Japan; ^19^ Division of Hematology, Department of Medicine Showa Medical University School of Medicine Tokyo Japan; ^20^ Department of Hematology Tokyo Metropolitan Institute for Geriatrics and Gerontology Tokyo Japan; ^21^ Department of Hematology Kyorin University Hospital Tokyo Japan; ^22^ Department of Hematology Japanese Red Cross Aichi Medical Center Nagoya Daiichi Hospital Nagoya Japan; ^23^ Central Japan Cord Blood Bank Seto Japan; ^24^ Japanese Red Cross Kanto‐Koshinetsu Block Blood Center Tokyo Japan; ^25^ Japanese Data Center for Hematopoietic Cell Transplantation Nagakute Japan; ^26^ Department of Registry Science for Transplant and Cellular Therapy Aichi Medical University School of Medicine Nagakute Japan; ^27^ Division of Hematology Jichi Medical University Saitama Medical Center Saitama Japan; ^28^ Division of Emerging Medicine for Integrated Therapeutics (EMIT), Center for Molecular Medicine Jichi Medical University Shimotsuke Japan; ^29^ Department of Hematology and Oncology Nagoya University Graduate School of Medicine Nagoya Japan

**Keywords:** cord blood transplantation, graft‐versus‐host disease, non‐relapse mortality, pre‐engraftment syndrome, relapse rate

## Abstract

Pre‐engraftment syndrome (PES) is a unique complication of cord blood transplantation (CBT) whose risk factors and impact on transplant outcomes remain controversial. Using a nationwide database in Japan, we analyzed a total of 3734 patients who underwent single‐unit CBT. PES occurred in 18.3% of patients, and risk factors for PES included a higher hematopoietic cell transplantation‐specific comorbidity index, first transplantation, myeloablative conditioning (MAC), lower total nucleated cell (TNC) dose, and graft‐versus‐host disease (GVHD) prophylaxis regimens excluding tacrolimus with methotrexate. Patients who developed PES had significantly higher incidences of grade II–IV acute GVHD (53.1% vs. 31.3%, *p* < 0.001) and chronic GVHD (27.2% vs. 21.7%, *p* = 0.002) compared to those without PES. Landmark analysis with multivariable adjustment revealed that PES was independently associated with increased non‐relapse mortality (NRM, hazard ratio [HR] 1.46; 95% CI 1.22–1.75; *p* < 0.001), reduced relapse incidence (HR 0.78; 95% CI 0.63–0.96; *p* = 0.020), and a trend toward inferior overall survival (OS, HR 1.13; 95% CI 0.98–1.30; *p* = 0.088). Moreover, patients who experienced both PES and acute GVHD had the highest 2‐year NRM (31.7%; 95% CI 26.0%–37.6%) and the lowest 2‐year OS (55.9%; 95% CI 50.0%–62.4%), compared with those who experienced either PES (NRM 20.7%; OS 65.0%) or acute GVHD (NRM 19.5%; OS 62.8%) (*p* < 0.001), highlighting the combined effects of these complications. This study, the largest to date on PES, demonstrates its clinical significance as an early complication with lasting effects on transplant outcomes.

## 1 Introduction

Umbilical cord blood (CB) offers unique advantages such as rapid availability and high tolerance to human leukocyte antigen (HLA) mismatch, making it a widely used alternative to allogeneic hematopoietic stem cell transplantation (allo‐HSCT) in patients without an HLA‐matched donor [1, 2]. Although high rates of graft failure and non‐relapse mortality (NRM) have historically been a major concern following cord blood transplantation (CBT), transplant outcomes have improved significantly over time [3]. Currently, more than 1300 CBT procedures are performed annually in Japan, with nearly all utilizing single‐unit CB, representing approximately one‐third of all CBTs performed worldwide [4].

A unique and early complication specific to CBT is pre‐engraftment syndrome (PES), also referred to as pre‐engraftment immune reaction (PIR) [4, 5, 6, 7, 8, 9, 10, 11, 12]. PES typically occurs within the first 2 weeks post‐transplant and is characterized by non‐infectious fever, skin rash, weight gain, peripheral edema, diarrhea, and liver dysfunction. Although the exact pathophysiology of PES remains unclear, it is hypothesized to result from donor T‐cell proliferation [13] and cytokine dysregulation [14], with interleukin‐6 (IL‐6) playing a central role in its development [15].

The reported incidence of PES varies widely, ranging from 20% to 78%, reflecting differences in diagnostic criteria, conditioning regimens, and graft‐versus‐host disease (GVHD) prophylaxis regimens [5, 6, 7, 8, 9, 10, 11, 12, 15]. The effect of PES on transplant outcomes has been controversial. While some retrospective studies have linked PES to an increased risk of acute and/or chronic GVHD [8, 9, 10], others have reported no such association [7, 11]. Most studies have found that PES does not significantly affect relapse rate, NRM, or overall survival (OS) [7, 8, 9, 10, 11], although a single‐center study linked PES to a lower relapse rate and better disease‐free survival in patients with myeloid malignancies [16].

To address and clarify these controversies, we conducted a large‐scale retrospective analysis of PES using data from the Japanese nationwide registry, the largest single‐unit CBT database globally [4]. This study aimed to investigate the incidence of PES, identify its risk factors, and evaluate its impact on transplant outcomes.

## 2 Methods

### 2.1 Study Design and Data Source

This retrospective study was conducted using the Transplant Registry Unified Management Program (TRUMP) database, maintained by the Japanese Society for Transplantation and Cellular Therapy (JSTCT) and the Japanese Data Center for Hematopoietic Cell Transplantation (JDCHCT) [17]. Written informed consent was obtained from all patients through their respective institutions. This study was approved by the TRUMP data management committee and the ethics committee of the Keio University School of Medicine, Tokyo, Japan.

### 2.2 Patients and Definitions

Based on the TRUMP database, this study included patients aged 16 years or older who underwent single‐unit CBT between 2019 and 2021. A diagnosis of PES was established according to the previously published definition [18] and based on non‐infectious fever and vascular leakage occurring more than 1 week before engraftment. The hematopoietic cell transplantation‐specific comorbidity index (HCT‐CI) was calculated using the established method [19]. Myeloablative conditioning (MAC) and reduced‐intensity conditioning (RIC) were defined according to the criteria of the Center for International Blood and Marrow Transplant Research (CIBMTR) [20, 21]. Neutrophil engraftment was defined as an absolute neutrophil count exceeding 0.5 × 10^9^/L for three consecutive days. Grading of acute GVHD was based on conventional criteria [22]. Standard‐risk diseases were defined as acute myeloid leukemia (AML) in first or second complete remission, acute lymphoblastic leukemia (ALL) in first complete remission, chronic myeloid leukemia (CML) in chronic or accelerated phase, low‐risk myelodysplastic syndrome (MDS), lymphomas with complete and partial response, and plasma cell neoplasms with complete, very good partial, or partial response. All other presentations of AML, ALL, CML, MDS, myeloproliferative neoplasms (MPNs), lymphomas, or plasma cell neoplasms in other conditions were classified as advanced‐risk diseases. HLA matching at the allele level between patient and donor was assessed by counting the mismatches at HLA‐A, ‐B, ‐C, and ‐DRB1.

### 2.3 Statistical Analysis

Continuous variables were summarized as medians with ranges or interquartile ranges (IQRs), and categorical variables were summarized as counts and percentages. Comparisons of categorical variables between two groups were performed using the chi‐squared test or Fisher's exact test, as appropriate. The probabilities of PES, acute GVHD, chronic GVHD, NRM, and relapse were estimated using the cumulative incidence function. For NRM, relapse was treated as a competing event for NRM, whereas for all other outcomes, death was treated as a competing event. The cumulative incidence of chronic GVHD was analyzed among patients who were alive and relapse‐free at day 90. Univariable and multivariable analyses were conducted using the Fine–Gray competing risk regression model to identify risk factors for the incidence of PES and acute GVHD, respectively. Covariates included in univariable and multivariable analyses were: recipient age at CBT, Eastern Cooperative Oncology Group (ECOG) performance status (PS), HCT‐CI, disease risk, prior transplantation, number of HLA allele mismatches, number of infused TNCs and CD34‐positive cells per kilogram of recipient body weight at transplantation, conditioning intensity, and GVHD prophylaxis. Variables with a *p*‐value of < 0.10 in the univariable analysis were included in the multivariable model. OS was estimated using the Kaplan–Meier method, and univariable comparisons of OS were performed using the log‐rank test. Patients who underwent subsequent transplantation were censored at the time of the next transplant.

A landmark analysis was performed to evaluate the impact of PES and acute GVHD on transplant outcomes. The landmark time point was set at day 16, by which time 95% of patients had developed PES. For the combined analysis of PES and acute GVHD, a second landmark was set at day 100.

All *p*‐values were two‐sided, and values < 0.05 were considered statistically significant. All statistical analyses were performed using R software (version 4.3.2).

## 3 Results

### 3.1 Patient and Transplant Characteristics

A total of 3734 patients were included in the analysis. Detailed patient characteristics are shown in Table 1. The median age at transplantation was 55 years (range 16–79), and the HCT‐CI scores were 28% intermediate risk (1–2) and 20% high risk (≥ 3). The most common underlying disease was AML (48%), followed by lymphoma (16%), ALL (15%), and MDS (14%). Disease risk was classified as standard in 45% of patients and advanced in 52%. HLA matching at the allele level revealed that 89% of patients had ≤ 4 mismatched alleles, while 11% had ≥ 5. The median infused TNC count was 2.73 (IQR 2.33–3.32) × 10^7^/kg. For conditioning intensity, 53% of patients received MAC, whereas 47% underwent RIC. For GVHD prophylaxis, 46% of patients received tacrolimus (TAC) plus mycophenolate mofetil (MMF), 34% received TAC plus methotrexate (MTX), 10% received cyclosporine (CsA) plus MTX, and 6% received TAC or CsA alone.

**TABLE 1:** Patient and transplant characteristics.

	Overall (*n* = 3734)
Sex	
Female	1595 (43%)
Male	2139 (57%)
*Age, years (range)*	55 (16–79)
< 55	1847 (49%)
≥ 55	1887 (51%)
ECOG PS	
0–1	3301 (89%)
≥ 2	427 (11%)
Unknown	6
HCT‐CI	
0	1932 (52%)
Intermediate risk (1–2)	1027 (28%)
High risk (≥ 3)	745 (20%)
Unknown	30
Diagnosis	
AML	1792 (48%)
MDS	530 (14%)
CML/MPN	129 (4%)
ALL	554 (15%)
Lymphoma	611 (16%)
Plasma cell neoplasm	22 (0.6%)
Others	96 (3%)
Disease risk	
Standard risk	1690 (45%)
Advanced risk	1929 (52%)
Bone marrow failure	33 (1%)
Others	82 (2%)
Number of transplants	
1	2876 (77%)
≥ 2	858 (23%)
No. of HLA allele mismatches	
0–1/8	320 (9%)
2/8	592 (17%)
3/8	1289 (37%)
4/8	892 (26%)
5/8	287 (8%)
≥ 6/8	117 (3%)
Unknown	237
*No. of infused TNCs (10* ^*7*^ */kg), median (IQR)*	2.73 (2.33–3.32)
≤ 2.5	1327 (36%)
> 2.5	2385 (64%)
Unknown	22
*No. of infused CD34‐positive cells (10* ^*5*^ */kg), median (IQR)*	0.86 (0.67–1.14)
≤ 0.75	1347 (36%)
> 0.75	2365 (64%)
Unknown	22
Conditioning intensity	
RIC	1739 (47%)
MAC	1995 (53%)
GVHD prophylaxis	
TAC + MTX	1274 (34%)
CsA + MTX	360 (10%)
TAC + MMF	1723 (46%)
CsA + MMF	99 (3%)
TAC/CsA alone	213 (6%)
Other	65 (2%)

Abbreviations: ALL, acute lymphoblastic leukemia; AML, acute myeloid leukemia; CML, chronic myeloid leukemia; CsA, cyclosporine; ECOG, Eastern Cooperative Oncology Group; GVHD, graft‐versus‐host disease; HCT‐CI, hematopoietic cell transplantation‐specific comorbidity index; HLA, human leukocyte antigen; IQR, interquartile range; MAC, myeloablative conditioning; MDS, myelodysplastic syndrome; MMF, mycophenolate mofetil; MPN, myeloproliferative neoplasms; MTX, methotrexate; PS, performance status; RIC, reduced‐intensity conditioning; TAC, tacrolimus; TNC, total infused nucleated cell.

### 3.2 Incidence and Risk Factors for PES

**FIGURE 1:**
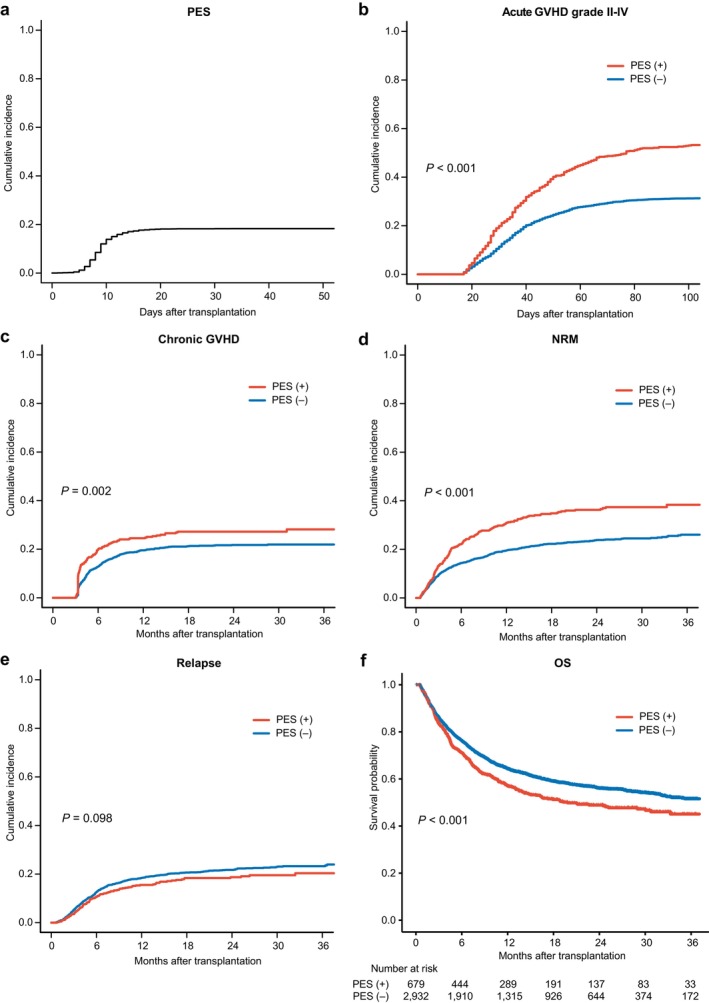
Cumulative incidences of PES and impact on transplant outcomes. (a) The cumulative incidence of PES in the entire cohort. The cumulative incidences of (b) grade II–IV acute GVHD, (c) chronic GVHD, (d) NRM, (e) relapse, as well as (f) the probability of OS, stratified by the presence or absence of PES. *p‐*Values are from univariable analyses using Cox proportional hazards regression for OS and Fine–Gray regression for GVHD, NRM, and relapse. [Color figure can be viewed at wileyonlinelibrary.com]

In the overall cohort, the cumulative incidence of PES was 18.3% (95% CI 17.0%–19.5%) at day 30 (Figure 1a). The median time to PES onset from transplantation was 9 days (IQR 7–10). In addition, the cumulative incidence of neutrophil engraftment was 82.0% (95% CI 80.7%–83.2%) by day 30, and the median time to neutrophil engraftment was 19 days (IQR 16–24) in the entire cohort (Figure S1).

In univariable competing risk analyses, intermediate‐ and high‐risk HCT‐CI, advanced‐risk disease, the use of MAC, ≤ 4 HLA allele mismatches, an infused TNC count < 2.5 × 10^7^/kg, and GVHD prophylaxis with TAC + MMF, TAC or CsA alone, and CsA + MTX were significantly associated with a higher incidence of PES (Table 2 and Figure S2). In the multivariable competing risk analysis, intermediate‐ and high‐risk HCT‐CI, first transplantation, the use of MAC, an infused TNC count of < 2.5 × 10^7^/kg, and GVHD prophylaxis with TAC + MMF, TAC or CsA alone, and CsA + MTX were significantly associated with a higher incidence of PES. Notably, the number of HLA mismatches did not remain significant after multivariable adjustment (Table 2).

**TABLE 2:** Univariable and multivariable analyses of risk factors for developing pre‐engraftment syndrome.

	Univariable			Multivariable		
	HR	95% CI	*p*	HR	95% CI	*p*
Age						
< 55	1					
≥ 55	1.00	0.86–1.16	0.970			
Sex						
Female	1					
Male	1.15	0.99–1.33	0.076	1.09	0.93–1.28	0.270
ECOG PS						
0–1	1					
≥ 2	1.10	0.87–1.37	0.430			
HCT‐CI						
0	1			1		
Intermediate risk (1–2)	1.45	1.21–1.72	< 0.001	1.43	1.20–1.70	< 0.001
High risk (≥ 3)	1.68	1.40–2.02	< 0.001	1.49	1.23–1.80	< 0.001
Disease risk						
Standard risk	1			1		
Advanced risk	1.35	1.16–1.57	< 0.001	1.08	0.92–1.26	0.340
Bone marrow failure	1.15	0.52–2.53	0.730	1.50	0.66–3.38	0.330
Other	0.93	0.52–1.67	0.820	0.92	0.48–1.78	0.810
Number of transplants						
1	1					
≥ 2	0.86	0.71–1.03	0.098	0.75	0.62–0.92	0.005
Conditioning intensity						
RIC	1			1		
MAC	1.70	1.46–1.99	< 0.001	1.54	1.31–1.82	< 0.001
No. of HLA allele mismatches						
≤ 4/8	1			1		
≥ 5/8	0.64	0.48–0.84	0.001	0.79	0.6–1.06	0.110
No. of infused TNCs (10^7^/kg)						
≤ 2.5	1			1		
> 2.5	0.65	0.56–0.75	< 0.001	0.72	0.62–0.84	< 0.001
No. of infused CD34^+^ cells (10^5^/kg)						
≤ 0.75	1					
> 0.75	1.10	0.94–1.29	0.230			
GVHD prophylaxis						
TAC + MTX	1			1		
CsA + MTX	4.84	3.27–7.15	< 0.001	4.24	2.82–6.38	< 0.001
TAC + MMF	10.47	7.67–14.29	< 0.001	9.96	7.20–13.77	< 0.001
CsA + MMF	1.86	0.80–4.34	0.150	1.85	0.78–4.38	0.160
TAC/CsA alone	10.73	7.26–15.85	< 0.001	11.62	7.71–17.51	< 0.001
Other	3.38	1.53–7.46	0.003	3.34	1.48–7.54	0.004

Abbreviations: CI, confidence interval; CsA, cyclosporine; GVHD, graft‐versus‐host disease; HCT‐CI, hematopoietic cell transplantation‐specific comorbidity index; HLA, human leukocyte antigen; HR, hazard ratio; MAC, myeloablative conditioning; MMF, mycophenolate mofetil; RIC, reduced‐intensity conditioning; TAC, tacrolimus; TNC, total infused nucleated cell.

### 3.3 Impact of PES on Transplant Outcomes

The median follow‐up for survivors was 16.8 months after CBT. Landmark analyses starting at day 16 showed that the cumulative incidence of grade II–IV acute GVHD at day 100 was significantly higher in patients with PES compared to those without it, at 53.1% (95% CI 49.1%–56.9%) vs. 31.3% (95% CI 29.5%–33.0%) (*p* < 0.001, Figure 1b). Similarly, the cumulative incidence of chronic GVHD at 2 years was also significantly higher in patients with PES, at 27.2% (95% CI 23.2%–31.4%) vs. 21.7% (95% CI 19.8%–23.7%) (*p* = 0.002, Figure 1c). In patients with PES, the NRM rates were 6.0% (95% CI 4.3%–8.0%) at day 50, 30.1% (95% CI 27.3%–34.7%) at 1 year, and 36.2% (95% CI 32.2%–40.3%) at 2 years, whereas patients without PES had NRM rates of 4.8% (95% CI 4.0%–5.6%) at day 50, 19.6% (95% CI 18.0%–21.2%) at 1 year, and 23.8% (95% CI 22.0%–25.7%) at 2 years (*p* < 0.001, Figure 1d). In contrast, the 2‐year cumulative incidence of relapse tended to be lower in patients with PES, at 18.7% (95% CI 15.6%–22.1%), compared to 21.7% (95% CI 20.0%–23.5%) in patients without PES, although the difference was not statistically significant (*p* = 0.098, Figure 1e). The estimated OS was significantly inferior in patients with PES compared to those without. In patients with PES, OS rates were 92.4% (95% CI 90.5%–94.5%) at day 50, 56.9% (95% CI 53.2%–61.0%) at 1 year, and 48.9% (95% CI 44.8%–53.3%) at 2 years. By contrast, in patients without PES, OS rates were 93.0% (95% CI 92.1%–93.9%) at day 50, 62.2% (95% CI 62.2%–66.0%) at 1 year, and 56.0% (95% CI 53.9%–58.1%) at 2 years (*p* < 0.001, Figure 1f). These findings suggest that while PES develops soon after transplantation, both NRM and OS gradually worsen after day 50 in patients with PES compared to those without.

To further clarify the independent impact of PES on transplant outcomes, we performed multivariable analyses including PES status and baseline factors. In these adjusted models, PES remained a significant predictor of increased NRM (HR 1.46, 95% CI 1.22–1.75; *p* < 0.001), was independently associated with a lower risk of relapse (HR 0.78, 95% CI 0.63–0.96; *p* = 0.020), and showed a trend toward inferior OS (HR 1.13, 95% CI 0.98–1.30; *p* = 0.088) (Table 3). These results confirm that, even after adjustment for differences in patient characteristics, PES increases NRM while modestly reducing the risk of relapse, resulting in a trend toward inferior OS.

**TABLE 3:** Univariable and multivariable analyses of transplant outcomes.

	NRM						Relapse						OS					
	Univariable			Multivariable			Univariable			Multivariable			Univariable			Multivariable		
	HR	95% CI	*p* ^a^	HR	95% CI	*p* ^a^	HR	95% CI	*p* ^a^	HR	95% CI	*p* ^a^	HR	95% CI	*p* ^a^	HR	95% CI	*p* ^a^
Age																		
< 55	1			1			1						1			1		
≥ 55	1.66	1.43–1.92	< 0.001	1.58	1.35–1.85	< 0.001	0.98	0.84–1.16	0.850				1.56	1.40–1.73	< 0.001	1.50	1.34–1.69	< 0.001
Sex																		
Female	1			1			1			1			1			1		
Male	1.43	1.23–1.65	< 0.001	1.37	1.18–1.59	< 0.001	1.20	1.02–1.42	0.027	1.22	1.02–1.45	0.027	1.44	1.29–1.61	< 0.001	1.37	1.23–1.54	< 0.001
ECOG PS																		
0–1	1			1			1						1			1		
≥ 2	2.54	2.08–3.09	< 0.001	2.06	1.68–2.53	< 0.001	1.20	0.92–1.56	0.180				2.66	2.31–3.05	< 0.001	2.14	1.85–2.47	< 0.001
HCT‐CI																		
0	1			1									1					
Intermediate risk (1–2)	1.27	1.07–1.51	0.007	1.07	0.90–1.27	0.460	0.89	0.73–1.08	0.240				1.17	1.04–1.33	0.012	1.00	0.88–1.13	0.940
High risk (≥ 3)	1.88	1.58–2.24	< 0.001	1.51	1.27–1.80	< 0.001	1.04	0.84–1.27	0.740				1.57	1.38–1.79	< 0.001	1.31	1.14–1.49	< 0.001
Disease risk																		
Standard risk	1			1			1			1			1			1		
Advanced risk	1.63	1.41–1.89	< 0.001	1.27	1.1–1.48	< 0.01	1.9	1.61–2.24	< 0.001	1.81	1.51–2.17	< 0.001	2.33	2.08–2.61	< 0.001	1.94	1.73–2.19	< 0.001
Bone marrow failure	1.07	0.47–2.44	0.870	1.25	0.56–2.77	0.580	NA	NA	NA	NA	NA	NA	0.68	0.30–1.51	0.278	0.68	0.28–1.66	0.403
Others	2.30	1.54–3.42	< 0.001	1.89	1.23–2.90	< 0.01	1.47	0.85–2.55	0.170	1.38	0.78–2.43	0.260	2.4	1.74–3.30	< 0.001	2.21	1.59–3.07	< 0.001
Number of transplants																		
1	1			1			1			1			1					
≥ 2	1.60	1.36–1.87	< 0.001	1.53	1.29–1.82	< 0.001	1.46	1.22–1.74	< 0.001	1.32	1.09–1.6	0.004	1.69	1.51–1.89	< 0.001	1.52	1.34–1.73	< 0.001
Conditioning intensity																		
RIC	1			1			1						1			1		
MAC	0.85	0.74–0.98	0.024	1.06	0.90–1.24	0.480	0.89	0.76–1.04	0.150				0.83	0.74–0.92	< 0.001	1.02	0.91–1.15	0.699
No. of HLA allele mismatches																		
≤ 4/8	1						1			1			1					
≥ 5/8	1.02	0.81–1.27	0.890				0.73	0.55–0.98	0.035	0.75	0.56–1.00	0.050	0.95	0.80–1.13	0.551			
No. of infused TNCs (10^7^/kg)																		
≤ 2.5	1						1						1					
> 2.5	0.93	0.81–1.08	0.360				1.12	0.94–1.32	0.200				1.00	0.90–1.12	0.947			
No. of infused CD34^+^ cells (10^5^/kg)																		
≤ 0.75	1			1			1			1			1			1		
> 0.75	0.80	0.69–0.92	0.002	0.85	0.73–0.98	0.026	1.19	1.00–1.41	0.046	1.23	1.03–1.48	0.024	0.9	0.80–1.00	0.048	0.94	0.84–1.05	0.281
GVHD prophylaxis																		
TAC + MTX	1			1			1						1			1		
CsA + MTX	1.31	1.01–1.69	0.04	1.30	1.00–1.70	0.047	0.92	0.68–1.24	0.570				1.20	0.99–1.45	0.071	1.22	1.00–1.49	0.051
TAC + MMF	1.53	1.29–1.82	< 0.001	1.06	0.88–1.27	0.550	1.00	0.83–1.19	0.970				1.3	1.15–1.47	< 0.001	1.08	0.95–1.23	0.257
CsA + MMF	1.84	1.19–2.85	0.006	1.43	0.92–2.23	0.120	0.63	0.34–1.15	0.130				1.44	1.05–1.98	0.024	1.23	0.89–1.70	0.210
TAC/CsA alone	2.30	1.72–3.07	< 0.001	1.54	1.13–2.09	0.006	0.84	0.58–1.24	0.390				1.84	1.49–2.28	< 0.001	1.42	1.13–1.78	0.003
Other	1.81	1.09–3.00	0.022	1.64	1.01–2.67	0.045	1.21	0.67–2.19	0.520				1.77	1.23–2.53	0.002	1.70	1.18–2.45	0.004
PES																		
Without PES	1			1			1			1			1			1		
With PES	1.64	1.41–1.93	< 0.001	1.46	1.22–1.75	< 0.001	0.84	0.68–1.03	0.098	0.78	0.63–0.96	0.020	1.24	1.09–1.41	< 0.001	1.13	0.98–1.30	0.088

Abbreviations: CI, confidence interval; CsA, cyclosporine A; ECOG PS, Eastern Cooperative Oncology Group performance status; GVHD, graft‐versus‐host disease; HCT‐CI, hematopoietic cell transplantation‐specific comorbidity index; HR, hazard ratio; MAC, myeloablative conditioning, HLA, human leukocyte antigen; MMF, mycophenolate mofetil; MTX, methotrexate; NA, not applicable; NRM, non‐relapse mortality; OS, overall survival; PES, pre‐engraftment syndrome; RIC, reduced‐intensity conditioning; TAC, tacrolimus; TNCs, total nucleated cells.

^a^ *p*‐Values were calculated using Cox proportional hazards regression for OS and Fine–Gray regression for NRM and relapse.

The causes of death in patients with and without PES are shown in Table S1. In patients without PES, the most common causes of death were relapse (43.5%), infection (17.3%), and organ failure (16.1%). In contrast, among patients with PES, relapse was less frequent (28.8%), while organ failure (27.6%) and infection (20.8%) were more common. A significant difference in the distribution of causes of death between the two groups was observed (*p* < 0.001).

### 3.4 Combined Effects of PES and Acute GVHD on Survival

**FIGURE 2:**
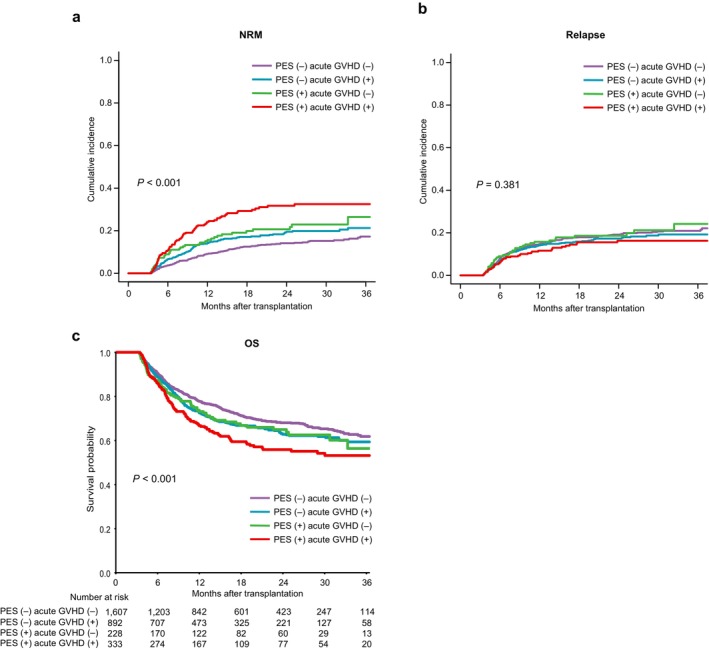
Transplant outcomes stratified by the development of PES and acute GVHD. The cumulative incidences of (a) NRM, (b) relapse, as well as (c) the probability of OS, stratified by the presence or absence of PES and grade II–IV acute GVHD. [Color figure can be viewed at wileyonlinelibrary.com]

As PES was frequently followed by the development of acute GVHD, the association of PES with higher NRM may be attributed to an increased risk of acute GVHD. Therefore, to separately assess the effects of PES and acute GVHD on transplant outcomes, we divided patients into four groups based on PES and grade II–IV acute GVHD, then performed landmark analysis for each group starting on day 100. The 2‐year cumulative NRM was higher in patients who developed either PES (20.7% [95% CI 14.9%–27.1%]) or acute GVHD (19.5% [95% CI 16.4%–22.9%]) compared to those without (14.2% [95% CI 12.0%–16.4%]). Remarkably, the highest NRM was observed in patients who developed both PES and acute GVHD, with a 31.7% 2‐year NRM (95% CI 26.0%–37.6%, *p* < 0.001, Figure 2a). In contrast, there were no significant differences in relapse rates among the four groups (*p* = 0.381, Figure 2b). Regarding OS, the 2‐year OS was lower in patients who developed either PES (65.0% [95% CI 58.1%–72.6%]) or acute GVHD (62.8% [95% CI 59.1%–66.7%]) compared to those without (68.0% [95% CI 65.3%–70.9%]). The worst outcomes were observed in patients who experienced both PES and acute GVHD, with a 2‐year OS of 55.9% (95% CI 50.0%–62.4%). These results, derived from unadjusted landmark analysis, suggest that the combination of PES and acute GVHD may be associated with particularly poor survival (*p* < 0.001, Figure 2c).

## 4 Discussion

This is the largest study to date of post‐CBT PES, offering valuable insight into its incidence, risk factors, and impact on transplant outcomes. The cumulative incidence of PES was 18.3%; risk factors for PES included intermediate‐ or high‐risk HCT‐CI, first transplantation, use of MAC, lower infused TNC count, and GVHD prophylaxis regimens other than TAC + MTX. PES was associated with higher rates of acute and chronic GVHD, a reduced risk of relapse, and an increased NRM, resulting in a tendency toward inferior OS. More importantly, patients who experienced both PES and acute GVHD had the worst outcomes, highlighting the combined effects of these complications. These findings suggest that PES is an important early complication with potential long‐term impact on transplant outcomes.

Consistent with prior studies [9, 10], our findings confirmed that MAC significantly increases the risk of PES. Intense conditioning regimens are known to cause tissue damage and trigger the release of pro‐inflammatory cytokines, which may drive the hyperimmune response associated with PES. In addition, previous studies have suggested that GVHD prophylaxis regimens without MTX are associated with a higher risk of PES [6, 10, 11]. Our study identified TAC + MTX as the combination associated with the lowest incidence of PES. CsA is associated with a higher rate of acute GVHD compared to TAC [23, 24], while MTX reduces the incidence of severe GVHD more than MMF [25], suggesting that TAC + MTX may be more immunosuppressive. Therefore, it is reasonable to conclude that TAC + MTX is the most effective combination for preventing the development of PES after CBT. TAC + MTX is widely used in Japan as GVHD prophylaxis after CBT and has been suggested to reduce NRM in this setting [26]. However, the optimal MTX dose after CBT remains undefined [27, 28, 29].

Although previous studies have shown that a higher infused TNC count increases the risk of PES [8, 10], a lower TNC count was associated with a higher incidence of PES in our analysis. These previous studies mainly included pediatric patients and adopted a threshold of 5 × 10^7^/kg or higher. In contrast, our study population consisted entirely of adults, and few patients (3%) received more than 5 × 10^7^/kg of TNC, which may account for the discrepancy between our results and those of previous studies. It is possible that massive expansion of infused hematopoietic cells may exaggerate the inflammatory response, leading to the development of PES, although the precise mechanism remains unknown. The association between PES and HCT‐CI has not been previously reported. However, a prior study has shown that a higher HCT‐CI is associated with an increased risk of acute GVHD [30]. Patients with high HCT‐CI may have chronic cytokine release and tissue damage due to pre‐existing comorbidities, which may trigger the development of PES. The mechanism underlying the increased risk of PES after the first transplant remains unclear. One possibility is that host antigen‐presenting cell reservoirs are more intact at the time of the first transplant, priming stronger early immune activation [31]. Regarding HLA matching, HLA allele‐level mismatch was not independently associated with PES after multivariable adjustment. Because CB units are typically selected with ≤ 2 antigen mismatches at HLA‐A/‐B/‐DR [32], any independent effect of residual allele‐level disparity may be limited and difficult to detect.

While previous studies have shown conflicting results regarding the association between PES and GVHD [7, 8, 9, 10, 11], our study clearly demonstrates a significant association between PES and both acute and chronic GVHD. This finding suggests overlapping risk factors and shared underlying mechanisms between PES and GVHD, such as tissue damage and the release of pro‐inflammatory cytokines [14, 33]. In addition, the intense systemic inflammatory response associated with PES may contribute to an environment conducive to GVHD development.

We also showed that PES significantly increased NRM, with a trend toward inferior OS. Most previous studies have concluded that PES does not affect NRM or OS [7, 8, 9, 10, 11]. However, one study showed that severe PES induced hemophagocytic syndrome and was associated with increased NRM [34], while another study showed that patients with PES tended to have higher NRM and worse OS than those without PES, although the difference was not statistically significant [11]. Our study clarified the prognostic impact of PES by using a large‐scale cohort and performing a landmark analysis to eliminate survivor bias, as well as multivariable analyses to account for differences in patient characteristics. Interestingly, the difference between NRM and OS across the two groups became more evident after 3 months (Figure 1d,f), suggesting that complications following PES, such as GVHD, organ dysfunction, and infections, rather than PES itself, were responsible for the prognosis. In addition, patients who developed both PES and acute GVHD had the worst survival, highlighting the additive risks of these complications.

The impact of PES on relapse has also been controversial. While many studies concluded that there was no effect, one study showed that PES was associated with a lower relapse rate in myeloid malignancies [16]. In our study, multivariable analysis of the entire cohort demonstrated that PES was independently associated with a reduced risk of relapse. This finding is consistent with a previous study showing that acute GVHD is associated with a reduced risk of relapse in CBT [35], supporting the idea that an early immune response may play an important role in the graft‐versus‐tumor (GVT) effect in CBT. Mechanistically, the GVT effect is thought to be driven predominantly by donor‐derived T cells that recognize recipient malignant cells through alloreactive responses and mediate cytotoxic clearance [36]; thus, the early immune activation that characterizes PES could plausibly amplify this T‐cell‐dependent effect. However, the significant association between PES and higher NRM underscores that a reduced risk of relapse does not result in improved survival.

Regarding the management of PES, systemic corticosteroids are the only established therapy [5]; however, a subset of patients develop steroid‐refractory severe PES with high early mortality [34]. Emerging translational and clinical data implicate monocyte‐derived IL‐6 in PES pathogenesis and suggest that IL‐6 blockade may reduce early NRM in steroid‐refractory cases [15]. Further studies are needed to define the optimal steroid dose and duration, as well as the timing and role of IL‐6 inhibition.

The limitations of this study include its retrospective nature. In addition, the database did not contain detailed clinical information such as the severity of PES or the dosage and duration of corticosteroid treatment. Moreover, the definition of PES varies among previous studies [5, 6, 7, 8, 9, 10, 11], which may affect comparisons between studies and the interpretation of our results. Whether the results of this study differ across ethnic groups or in the context of double‐unit CBT requires further investigation [37, 38].

In conclusion, this study demonstrated that PES significantly impacts transplant outcomes following CBT and identified PES risk factors. In particular, we showed the association of PES with increased NRM, reduced relapse rates, and a trend toward inferior OS.

## Author Contributions

M.S. and K.K. designed the study, performed the analyses, and wrote the manuscript. K.M. assisted with the statistical analyses. T.M., S.S., H.S., T.M., and J.K. assisted in interpreting the results. M.T., M.T., M.O., M.S.‐Y., J.I., Y.K., S.O., M.S., J.K., Y.H., K.O., N.H., S.M., N.T., T.N., K.K., and F.I. provided clinical data. Y.A. managed the unified registry database and revised the manuscript. S.T. and H.N. advised on the methods, revised the manuscript, and were responsible for the co‐project leads of JSTCT GVHD and JSTCT Transplant Complication Working Groups, respectively. All authors discussed the results and reviewed the manuscript.

## Conflicts of Interest

Masatoshi Sakurai owns stock in Celaid Therapeutics, outside the submitted work. Keisuke Kataoka received honoraria from Ono Pharmaceutical, Eisai, Astellas Pharma, Novartis, Chugai Pharmaceutical, AstraZeneca, Sumitomo Pharma, Kyowa Kirin, Janssen Pharmaceutical, Takeda Pharmaceutical, Otsuka Pharmaceutical, SymBio Pharmaceuticals, Bristol Myers Squibb, Pfizer, Nippon Shinyaku, Daiichi Sankyo, Alexion Pharmaceuticals, AbbVie, Meiji Seika Pharma, Sanofi, Sysmex, Mundipharma, Incyte Corporation, and Kyorin Pharmaceutical; received research support from Otsuka Pharmaceutical, Chordia Therapeutics, Chugai Pharmaceutical, Takeda Pharmaceutical, and Meiji Seika Pharma; received scholarships from Asahi Kasei Pharma, Eisai, Otsuka Pharmaceutical, Ono Pharmaceutical, Kyowa Kirin, Shionogi, Takeda Pharmaceutical, Sumitomo Dainippon Pharma, Chugai Pharmaceutical, Teijin Pharma, Japan Blood Products Organization, Mochida Pharmaceutical, JCR Pharmaceuticals, and Nippon Shinyaku; owns stock in Asahi Genomics; and holds patents for “Genetic alterations as a biomarker in T‐cell lymphomas” and “PD‐L1 abnormalities as a predictive biomarker for immune checkpoint blockade therapy”, outside the submitted work. Shuichi Shirane received research support from PharmaEssentia Japan K.K., outside the submitted work. Takehiko Mori reports personal fees from Asahi Kasei Pharma, outside the submitted work. Masashi Sawa reports personal fees from Kyowa Kirin, Chugai Pharmaceutical, Pfizer, Astellas Pharma, Nippon Shinyaku, Ono Pharmaceutical, MSD, Bristol Myers Squibb, Asahi Kasei Pharma, Novartis, Eisai, Otsuka Pharmaceutical, Sumitomo Dainippon Pharma, Sanofi, Takeda Pharmaceutical, Mundipharma, AbbVie, CSL Behring, SymBio Pharmaceuticals, Janssen Pharmaceutical, AstraZeneca, Daiichi Sankyo, Amgen, Novo Nordisk, and Nippon Kayaku, outside the submitted work. Koji Kato received research support from Otsuka Pharmaceutical, Pfizer, AbbVie, Celgene, and MSD, outside the submitted work. Hideki Nakasone has received subsidies from JCR Pharmaceutical, Kyowa Kirin, Taiho Pharma, Santen Pharmaceutical, and Terumo, and honoraria from MSD, Otsuka Pharmaceutical, Novartis, Takeda Pharmaceutical, Janssen Pharmaceutical, Chugai Pharmaceutical, Sanofi, Meiji Seika Pharma, and Asahi Kasei Pharma, outside the submitted work. The other authors declare no competing interests.

## Supporting information


**Figure S1:** Cumulative incidence of engraftment
The cumulative incidence of neutrophil engraftment in the entire cohort.

**Figure S2:** Cumulative incidences of PES according to each variable
The cumulative incidences of PES stratified by (a) HCT‐CI, (b) disease risk, (c) conditioning intensity, (d) the number of HLA allele mismatches, (e) the number of infused TNC, and (f) GVHD prophylaxis regimens.

**Table S1:** Causes of death in patients with and without pre‐engraftment syndrome.

## Data Availability

The data of this study are not publicly available due to ethical restrictions because sharing would exceed the scope of the recipient/donor's consent for research use in the registry.
