# Supplementary figures and images for: Risk Factors for and Impact of Pre‐Engraftment Syndrome on Outcomes Following Single‐Unit Cord Blood Transplantation in Adults
Source: Am J Hematol. 2025 Sep 26;100(12):2248–60. doi: 10.1002/ajh.70094 (PMC12603891; doi:10.1002/ajh.70094)

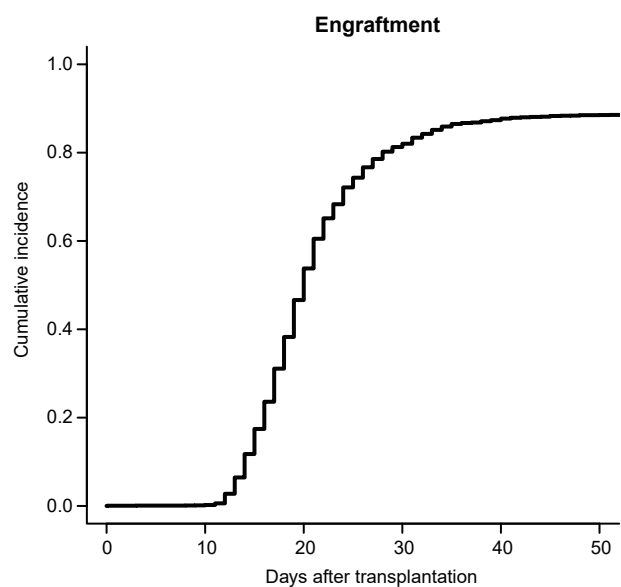

Supplement: Supplementary file 1 — Figure S1: Cumulative incidence of engraftment The cumulative incidence of neutrophil engraftment in the entire cohort. [file AJH-100-2248-s001.pdf]

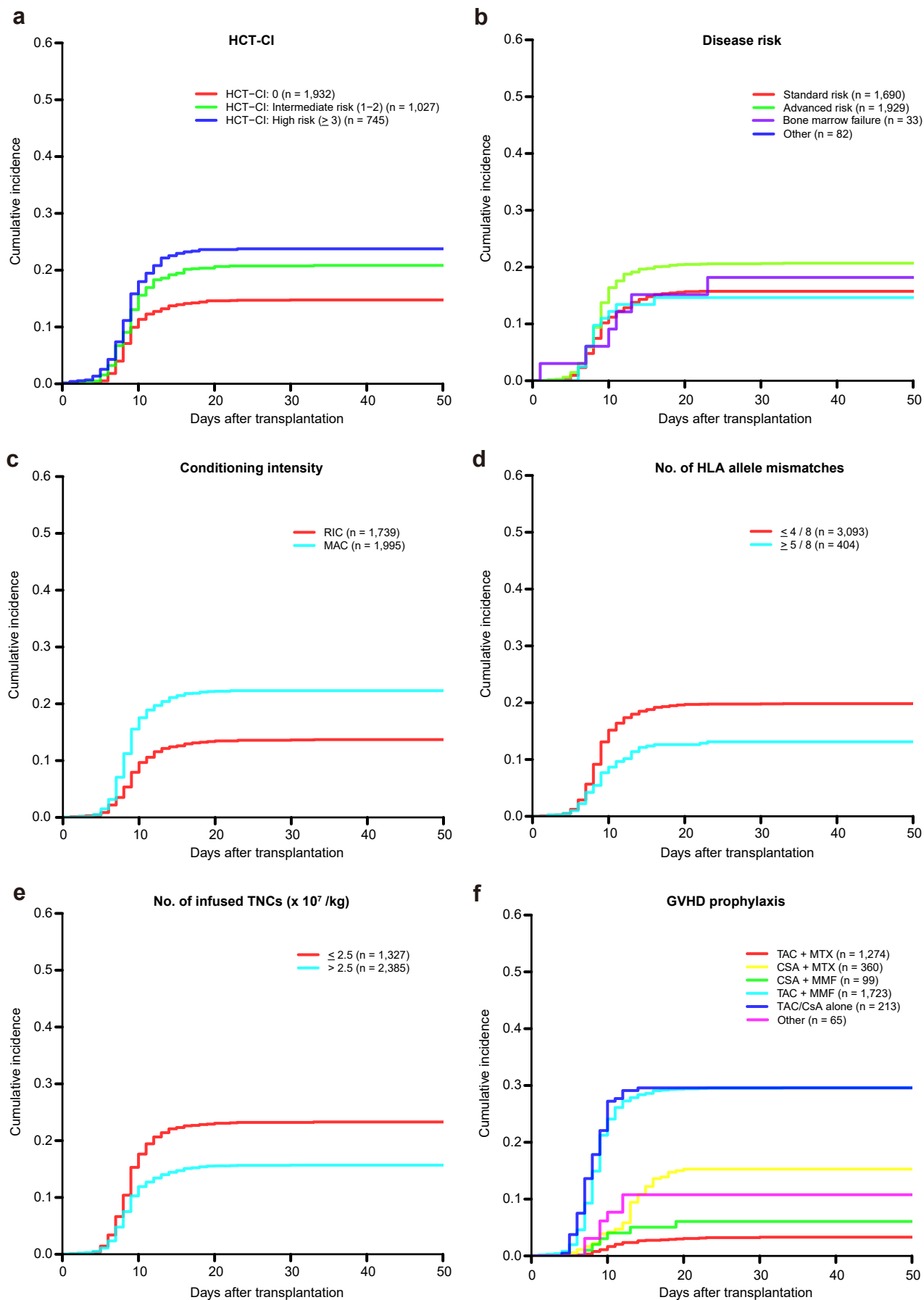

**Supplementary Figure 2**

Supplement: Supplementary file 2 — Figure S2: Cumulative incidences of PES according to each variable The cumulative incidences of PES stratified by (a) HCT‐CI, (b) disease risk, (c) conditioning intensity, (d) the number of HLA allele mismatches, (e) the number of infused TNC, and (f) GVHD prophylaxis regimens. [file AJH-100-2248-s002.pdf]
