# Supplementary material for: Risk Factors for and Impact of Pre‐Engraftment Syndrome on Outcomes Following Single‐Unit Cord Blood Transplantation in Adults
Source: Am J Hematol. 2025 Sep 26;100(12):2248–60. doi: 10.1002/ajh.70094 (PMC12603891; doi:10.1002/ajh.70094)
Supplement: Supplementary file 3 — Table S1: Causes of death in patients with and without pre‐engraftment syndrome. [file AJH-100-2248-s003.docx]

| **Supplementary Table 1. Causes of death in patients with and without pre-engraftment syndrome** | | | |
| --- | --- | --- | --- |
|  | **Without PES n (%)** | **With PES n (%)** |  |
| Relapse | 521 (43.0%) | 94 (27.8%) |  |
| Infection | 213 (17.6%) | 71 (21.0%) |  |
| Organ failure | 196 (16.2%) | 93 (27.5%) |  |
| GVHD | 63 (5.2%) | 25 (7.4%) |  |
| SOS/VOD | 49 (4.0%) | 25 (7.4%) |  |
| Other | 169 (14.0%) | 30 (8.9%) | *P* < 0.001 |
| PES, pre-engraftment syndrome; GVHD, graft-versus-host disease; SOS/VOD, sinusoidal obstruction syndrome/veno-occlusive disease | | | |
